# Exploring the Therapeutic Potential of Scorpion-Derived Css54 Peptide Against *Candida albicans*

**DOI:** 10.1007/s12275-024-00113-4

**Published:** 2024-04-08

**Authors:** Jonggwan Park, Hyeongsun Kim, Da Dam Kang, Yoonkyung Park

**Affiliations:** 1 Department of Bioinformatics, Kongju National University, Kongju, 32588 Republic of Korea; 2 Department of Biomedical Science, Chosun University, Gwangju, 61452 Republic of Korea

**Keywords:** Antimicrobial peptide, *Candida albicans*, Antifungal activity, Antifungal mechanism

## Abstract

*Candida albicans* (*C. albicans*) is one of the most common opportunistic fungi worldwide, which is associated with a high mortality rate. Despite treatment, *C. albicans* remains the leading cause of life-threatening invasive infections. Consequently, antimicrobial peptides (AMPs) are potential alternatives as antifungal agents with excellent antifungal activity. We previously reported that Css54, found in the venom of *Centrurodies suffusus suffusus* (*C. s. suffusus*) showed antibacterial activity against zoonotic bacteria. However, the antifungal activity of Css54 has not yet been elucidated. The objective of this study was to identify the antifungal activity of Css54 against *C. albicans* and analyze its mechanism. Css54 showed high antifungal activity against *C. albicans*. Css54 also inhibited biofilm formation in fluconazole-resistant fungi. The antifungal mechanism of action of Css54 was investigated using membrane-related assays, including the membrane depolarization assay and analysis of the membrane integrity of *C. albicans* after treatment with Css54. Css54 induced reactive oxygen species (ROS) production in *C. albicans*, which affected its antifungal activity. Our results indicate that Css54 causes membrane damage in *C. albicans*, highlighting its value as a potential therapeutic agent against *C. albicans* infection.

**Supplementary Information:**

The online version contains supplementary material available at 10.1007/s12275-024-00113-4.

## Introduction

Global health challenges persist despite medical advancements because pathogenic microorganisms show increased resistance to conventional antimicrobial agents. These challenges have resulted in an increase in infectious diseases and mortality rates worldwide (Madanchi et al., 2022). Therefore, sustained exposure of humans and animals to resistant microorganisms can cause serious infections (Van Eijk et al., 2020).

Infections caused by drug-resistant fungi have become increasingly important clinical issues. Several factors contribute to these clinical issues, including surgical procedures and immunosuppression (Berman & Krysan, 2020). Infections with drug-resistant fungi can be difficult to treat, with many antifungal drugs ceasing to be effective (Bhattacharyya et al., 2020). People with weakened immune systems are at a particularly high risk of mortality when infected with drug-resistant fungi (Garvey et al., 2022). Thus, patients need to stay in hospital longer to improve their chance of survival. Therefore, the development of novel antifungal agents that inhibit drug resistance is essential.

Human fungal infections are predominantly caused by *Candida* spp (Lopes & Lionakis, 2022). *Candida* strains that demonstrate resistance to primary antifungal agents, including echinocandins and azoles, are progressively emerging (Pappas et al., 2018). Among the most common fungal pathogens, *Candida albicans* (*C. albicans*) causes invasive infections associated with high mortality rates. *Candida albicans* is the leading cause of healthcare-associated bloodstream infections in the United States, and patients with this infection encounter a mortality rate of 40% despite receiving antifungal treatment (Lee et al., 2020). In healthy individuals, *C. albicans* is a harmless commensal fungi that can be found in the oral cavity and gastrointestinal tract. However, in immunosuppressed patients, *C. albicans* can easily disseminate into the blood and invade organs, causing fatal systemic infections (Bertolini et al., 2019).

Antimicrobial peptides (AMPs), which are naturally produced by various organisms, exhibit substantial potential as viable substitutes for antibiotics to treat a diverse spectrum of infectious diseases (do Nascimento Dias et al., 2020). AMPs serve as the primary defense against microbial infections and have attracted significant attention as promising candidates for the development of novel antifungal treatments owing to their minimal toxicity and low rates of resistance (Buda De Cesare et al., 2020). Venoms synthesized by species such as scorpions, snakes, and spiders serve both defensive and predatory functions. AMPs from scorpion venom exhibit various antibacterial, anticancer, and anti-inflammatory properties (Nasr et al., 2023).

Css54 is a 25-amino acid α-helix peptide that was first isolated from *Centruroides suffuses suffuses* (*C. s. suffuses*) (Garcia et al., 2013). We previously demonstrated that Css54 exhibits antimicrobial activity against both Gram-positive and Gram-negative bacteria. Additionally, Css54 exhibited stable antibacterial activity under various pH and salt conditions. Moreover, we demonstrated that Css54 exhibits antimicrobial activity by disrupting the bacterial membrane (Park et al., 2020). However, the antifungal activity and mechanism of action of Css54 are poorly understood.

This study is the first to investigate the antifungal activity of the antimicrobial peptide Css54 against *C. albicans*. Css54 exhibits antifungal activity against *C. albicans* through membrane-disruptive mechanisms and driving excessive formation of reactive oxygen species (ROS). These characteristics of Css54 suggest that it may be a viable option for treating infections caused by *C. albicans*.

## Materials and Methods

### Materials

Yeast extract peptone dextrose (YPD) broth, and crystal violet were purchased from LPS Solution; bis (1,3-dibutylbarbituric acid) trimethine oxonol (DiBAC_4_[3]), SYTOX green, propidium iodide (PI), 2, 7-dichlorofluorescin diacetate (DCFH-DA), N-acetyl cysteine (NAC), fluconazole, and amphotericin B were purchased from Merck; mannan from *Saccharomyces cerevisiae* and laminarin from *Laminaria digitata* were purchased from Merck. RPMI medium was purchased from Welgene.

### Microorganisms

The reference strain, *C. albicans* (Korea Collection for Type Culture [KCTC] 7270), isolated from a human skin lesion in Uruguay, was acquired from the KCTC. The drug-resistant *C. albicans* strains (Culture Collection of Antibiotic-Resistant Microbes [CCARM]) 14,001, CCARM 14004, and CCARM 14007 from 1999, and CCARM 14020 from 2002 were sourced from CCARM.

### Peptide Synthesis

Peptides were synthesized via the solid-phase F-moc method on a Rink amide-4-methylbenzhydrylamine resin using a Liberty microwave peptide synthesizer (CEM Co.) (Park et al., 2020). Linkage reagents included 0.1 M hydroxybenzotriazole in piperidine/dimethylformamide and 0.45 M 2-(1H-benzotriazole-1-yil)-1,1,3,3-tetramethyluroniunm hexafluorophosphate in dimethylformamide. Following dichloromethane washes, cleavage occurred in a trifluoroacetic acid, phenol, water, and triisopropylsilane mixture for 2 h at 25 °C. Crude peptides were precipitated using ice-cold ether, dried, reconstituted in distilled water, and purified by RP-HPLC on a Jupiter C18 column (Phenomenex). Peptide molecular weights were determined by Matrix-Assisted Laser Desorption/Ionization—Time of Flight Mass Spectrometry (MALDI-TOF MS) (Kratos Analytical, Inc.).

### Antifungal Activity

The broth microdilution method, albeit with modifications, was employed to confirm the minimum inhibitory concentrations (MICs) of Css54 and fluconazole (Radhakrishnan et al., 2018). Css54 and antifungal agents were serially diluted in 10 mM phosphate buffer saline (PBS) and plated on 96-well plates. Subsequently, *C. albicans* (2 × 10^4^ CFU/ml) cultured overnight at 28 °C in YPD media and RPMI medium, was added to the plates and incubated for 16 h. The growth of *C. albicans* was quantified by measuring the absorbance at 600 nm using a Spectra Ma × M3 microplate reader (Molecular Devices). The MICs were established as the minimum concentration at which the peptides or antifungal agents inhibited 90% of the growth relative to the control. An aliquot from each well was plated onto YPD agar. Following incubation, the minimum fungicidal concentration (MFC) was determined to be the lowest concentration of a peptide and antifungal agent, yielding no observable growth (Snyder et al., 2021).

### Time-Kill Kinetics

The antifungal activity of Css54 against *C. albicans* KCTC 7270 and *C. albicans* CCARM 14020 was quantified by evaluating the CFU at specific incubation intervals (Dong et al., 2018). *Candida albicans* was standardized to 2 × 10^4^ CFU/ml and treated with Css54 1 × MIC and 2 × MIC and incubated at 28 °C. At specific time points, the suspensions were plated onto YPD agar and incubated for 24 h, before counting the colonies. The survival rate was determined using the following formula:

$${\text{Survival rate }}\left( \% \right) = \left( {{\text{CFU of}}\,C. \, albicans\,{\text{in Css54 solution}} - {\text{CFU in control solution }}/{\text{ CFU in control solution}}} \right) \times 100$$

### Biofilm Inhibition Assays

To analyze biofilm inhibition, *C. albicans* (KCTC 7270 and CCARM 14020) suspensions (1 × 10^6^ cells/ml in RPMI with 2% glucose) and varying peptide concentrations (0.5–8 μM) were combined in polystyrene 96-wells plates and incubated at 28 °C for 24 h (Aguiar et al., 2020). The supernatant was discarded and the biofilms were fixed with methanol for 10 min. After drying at room temperature, the biofilms were stained with 0.1% crystal violet for 30 min, before rinsing the stain with distilled water until the control was clear. Stained biofilms were solubilized in 95% ethanol and the absorbance at 595 nm was read using a Spectra Ma × M3 microplate reader (Molecular Devices) (Subramaniyan et al., 2023). The percentage of biofilm mass was determined using the following formula: (absorbance (A) _595 nm_ of treated/A_595 nm_ of control) × 100.

### Interaction of Css54 with Polysaccharide by CD (Circular dichroism) Spectroscopy

The structure of Css54 was evaluated in the PBS containing mannan and laminarin. The peptide was prepared at 40 μM in the presence of mannan and laminarin at (0.1–0.4% in PBS). CD measurements were performed using a JASCO 810 spectropolarimeter. Spectra were recorded at wavelengths of 190–250 nm in a 1.0 mm quartz cell (Han et al., 2016; Oh et al., 2022).

### Interaction of Css54 with Fungal Wall Components

The binding efficacy of Css54 to fungal cell wall components was evaluated by analyzing its effect on the antifungal activity of Css54. Css54 at the 2 × MIC was incubated with different concentrations of mannan and laminarin. The concentration range for mannan and laminarin was 0.003–4 mg/ml. After incubation for 16 h at 28 °C, the growth of *C. albicans* in the presence of fungal cell wall components and Css54 was measured at an absorbance of 600 nm (Han et al., 2016; Ramamourthy et al., 2020).

### Effect of Css54 on Membrane Potential

The change in the membrane potential of *C. albicans* after treatment with Css54 was assessed using DiBAC_4_(3) (Kim & Lee, 2019; You et al., 2020). *Candida albicans* (KCTC 7270) was washed three times with PBS. *Candida albicans* (4 × 10^7^ CFU/ml) in PBS was treated with Css54 at 0.25 × , 0.5 × , 1 × , and 2 × MIC for 2 h at 28 °C. *Candida albicans* was harvested by centrifugation and diluted in PBS. *Candida albicans* was exposed to DiBAC4(3) (2 μg/ml) for 10 min. The fluorescence intensity of DiBAC_4_(3) was assessed by flow cytometry (Beckman).

### PI Membrane Integrity Assay

The PI uptake assay was used to investigate the membrane integrity of *C. albicans* (KCTC 7270) after treatment with Css54. *Candida albicans* (4 × 10^7^ CFU/ml) in PBS was exposed to Css54 at concentrations of 0.25 × , 0.5 × , 1 × and 2 × MIC and incubated at 28 °C for 10 min. Subsequently, *C. albicans* cells were washed with PBS. PI (10 μg/ml) was added to *C. albicans* and reacted for 10 min. Subsequently, *C. albicans* was washed with PBS to eliminate unbound PI, and the fluorescence intensity was determined using flow cytometry (Beckman Coulter). The fluorescence intensity of PI was confirmed using an EVOS FL Auto 2 imaging system (Invitrogen) at 10 min.

### SYTOX Green Assay

SYTOX Green was used to further investigate the membrane damage in *C. albicans* after treatment with Css54. *Candida albicans* (KCTC 7270) was suspended in PBS supplemented with SYTOX Green (1 μM) and treated with Css54 at 0.25 × , 0.5 × , 1 × , and 2 × MICs. The fluorescence of SYTOX Green was measured for 30 min at a wavelength of 485 nm and an emission wavelength of 520 nm at 5 min intervals.

### Css54-Induced ROS Generation by *C. albicans*

Intracellular ROS levels were quantified using a fluorometric assay with DCFH-DA. *Candida albicans* cells (2 × 10^7^ CFU/ml) were treated with Css54 at 0.25, 0.5, 1, or 2 × MICs. DCFH-DA fluorescence intensity was determined by flow cytometry (Beckman Coulter). Css54 (1 × MIC was co-incubated with NAC (5, 7, and 10 mM) and *C. albicans* (2 × 10^4^ CFU/ml). After incubation for 16 h, the growth of *C. albicans* was assessed by measuring the absorbance at 600 nm.

## Results

### Antifungal Activity of Css54 Against *C. albicans*

The sequences, molecular weights, and toxicities of the Css54 peptides are summarized in Table 1. Css54 comprises 25 amino acids. At a concentration of 5 μM, the cytotoxicity of Css54 was 53.92%, while the hemolysis at 4 μM was 2.65% (Table 1). The antifungal activities of Css54 and fluconazole against five *C. albicans* strains in YPD Table 2. Css54 had MICs and MFCs of 2 μM and 4 μM in non-resistant *C. albicans* (KCTC 7270). Css54 exhibited consistent MIC and MFC values of 2 μM and 4 μM, respectively, against four strains of fluconazole-resistant *C. albicans* (CCARM 14001, 14,004, 14,007, 14,020) in YPD. Fluconazole exhibited MIC and MFC values of 16 μM and 32 μM, respectively against *C. albicans* (KCTC 7270). However, for the other four strains of *C. albicans*, fluconazole did not demonstrate antifungal activity up to a concentration of 256 μM. We also conducted an experiment to evaluate the impact of Css54 peptide treatment on *C. albicans* survival in RPMI medium. When treated with 2 µM and 4 µM of Css54, the survival rate of *C. albicans* in RPMI medium decreased (Fig. S1).

**Table 1:** Peptide characterization and cytotoxicity of Css54

Peptide	Sequence		MW^a^ (Da)	Cytotoxicity	
				Cell survial rate^b^ (%)	Hemolysis^c^ (%)
Css54	FFGSLLSLGSKLLPSVFKLFQRKKE-NH_2_		2869.5	53.92%	2.65%

^a^MW (molecular weight)

^b^Survival percentage of PK(15) cells, isolated from porcine kidney was assessed following treatment 5 μM Css54

^c^Hemolysis percentage in sheep red blood cells exposed to 4 μM Css54 was evaluated (Park et al., 2020)

**Table 2:** Antifungal activity of Css54 and fluconazole against *C. albicans* in YPD

Strains	MICs (μM)			
	Css54		Fluconazole	
	MIC	MFC	MIC	MFC
*C. albicans* (KCTC 7270)	2	4	16	32
*C. albicans* (CCARM 14001)	2	4	ND^a^	> 256
*C. albicans* (CCARM 14004)	2	4	ND	> 256
*C. albicans* (CCARM 14007)	2	4	ND	> 256
*C. albicans* (CCARM 14020)	2	4	ND	> 256

^a^ND (not detected)

### Time-Killing Assay

**Fig. 1:**
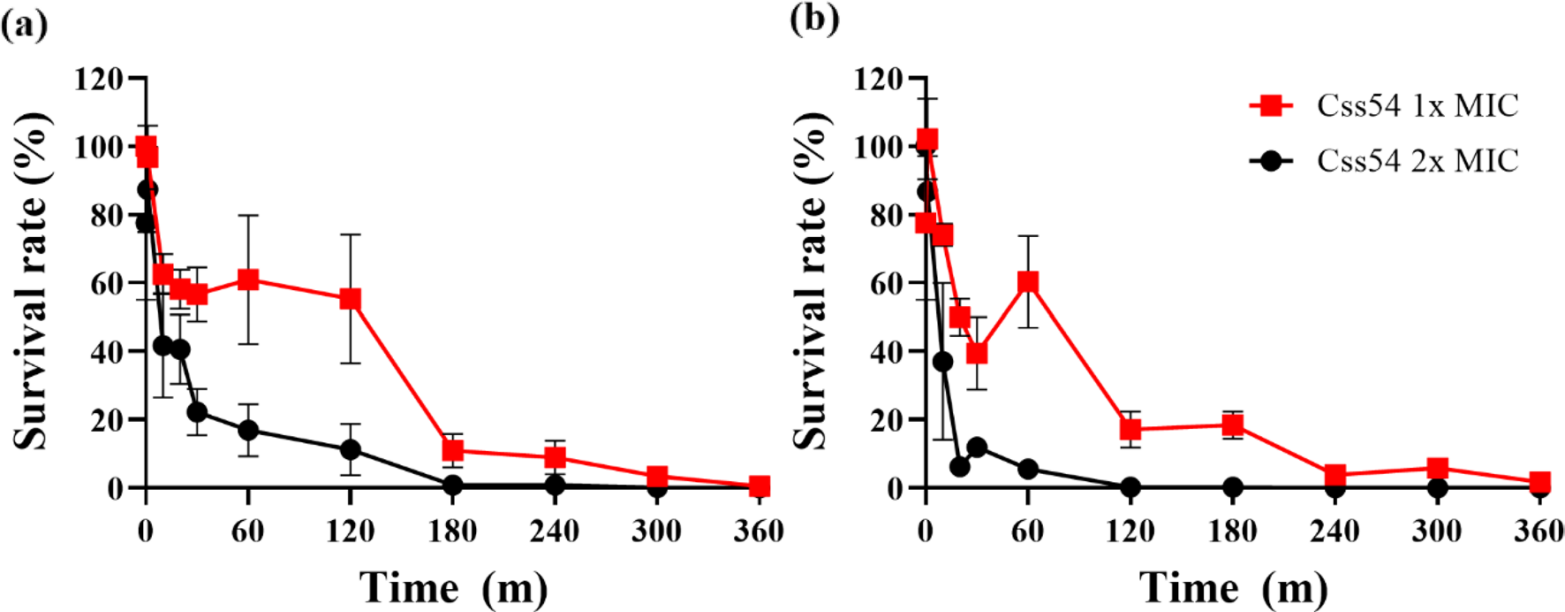
Time-killing kinetics of Css54 against *C. albicans*. **A** *C. albicans* (KCTC 7270) and **B** *C. albicans* (CCARM 14020) were treated with Css54 at 1 × MIC and 2 × MIC. Samples were plated on YPD agar at specified time intervals at specified time intervals and incubated at 28 °C. Data are shown as mean ± SEM (n = 4)

Time-killing assays of Css54 at 1 × MIC and 2 × MIC were performed for *C. albicans* KCTC 7270 and *C. albicans* CCARM 14020, respectively. After 3 h, Css54 (1 × MIC reduced *C. albicans* KCTC7270 by more than 20%. Css54 at 2 × MIC perfectly killed *C. albicans* KTC 7270 cells after 6 h (Fig. 1A). After 5 h, Css54 at 1 × MIC reduced the fluconazole resistant *C. albicans* strain CCARM 14020 by more than 20%. Css54 at the 2 × MIC effectively killed *C. albicans* CCARM 14020 after 3 h (Fig. 1B). These results show that Css54 exerts its antifungal activity in a dose-dependent manner.

### Biofilm Inhibition Assay

**Fig. 2:**
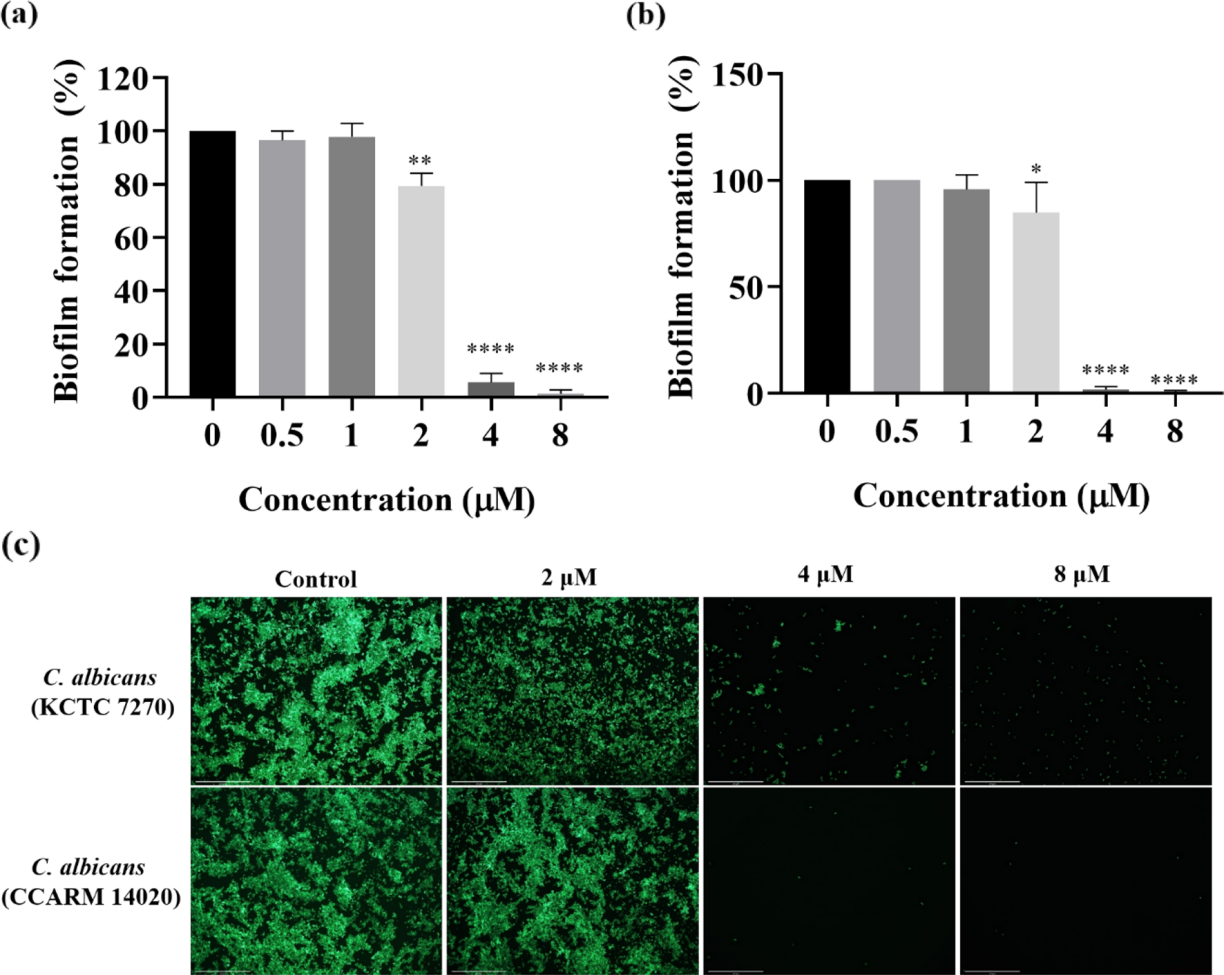
Investigation of the anti-biofilm activities of Css54. Css54 peptide inhibits biofilm formation by **A** *C. albicans* (KCTC 7270) and **B** *C. albicans* (CCARM 14020). Using crystal violet staining and measuring absorbance at 595 nm, the mass of the biofilm was determined. MBIC values were determined according to the materials and methods section. **C** *C. albicans* in biofilms were visualized using fluorescence microscopy and the effects of exposure to Css54. Live *C. albicans* in biofilms were stained with SYTO9. Data are shown as mean ± SEM (n = 6). *P < 0.05, **P < 0.01, ***P < 0.001, ****P < 0.0001versus control (unpaired *t-test*)

To assess the inhibitory effect of Css54 on biofilm formation by *C. albicans*, biofilm-forming strains were evaluated in RPMI supplemented with glucose. We previously confirmed that among the *C. albicans* strains, KCTC 7270 and CCARM 14020 formed biofilms (Ramamourthy et al., 2020). Using crystal violet staining, we demonstrated that Css54 at 4 μM inhibited more than 90% of the biofilm formation by both *C. albicans* strains (Fig. 2A, B). The value that inhibited biofilm formation by more than 90% was set as the minimum inhibitory concentration (MBIC). For both strains, the MBIC value of CSS54 was determined to be 4 μM. To visualize *C. albicans* in biofilms, we used SYTO9, a green fluorescent live cell staining dye to visualize live *C. albicans* in biofilm and analyzed the viability of *C. albicans* after exposure to Css54 at 0.5 × , 1 × , and 2 × MBIC. Css54 at 1 × MBIC significantly inhibited viability of *C. albicans* in biofilms. These results showed that Css54 significantly impacted biofilm formation by *C. albicans* (Fig. 2C).

### Interaction of Css54 with Polysaccharides of the Fungal Membrane

**Fig. 3:**
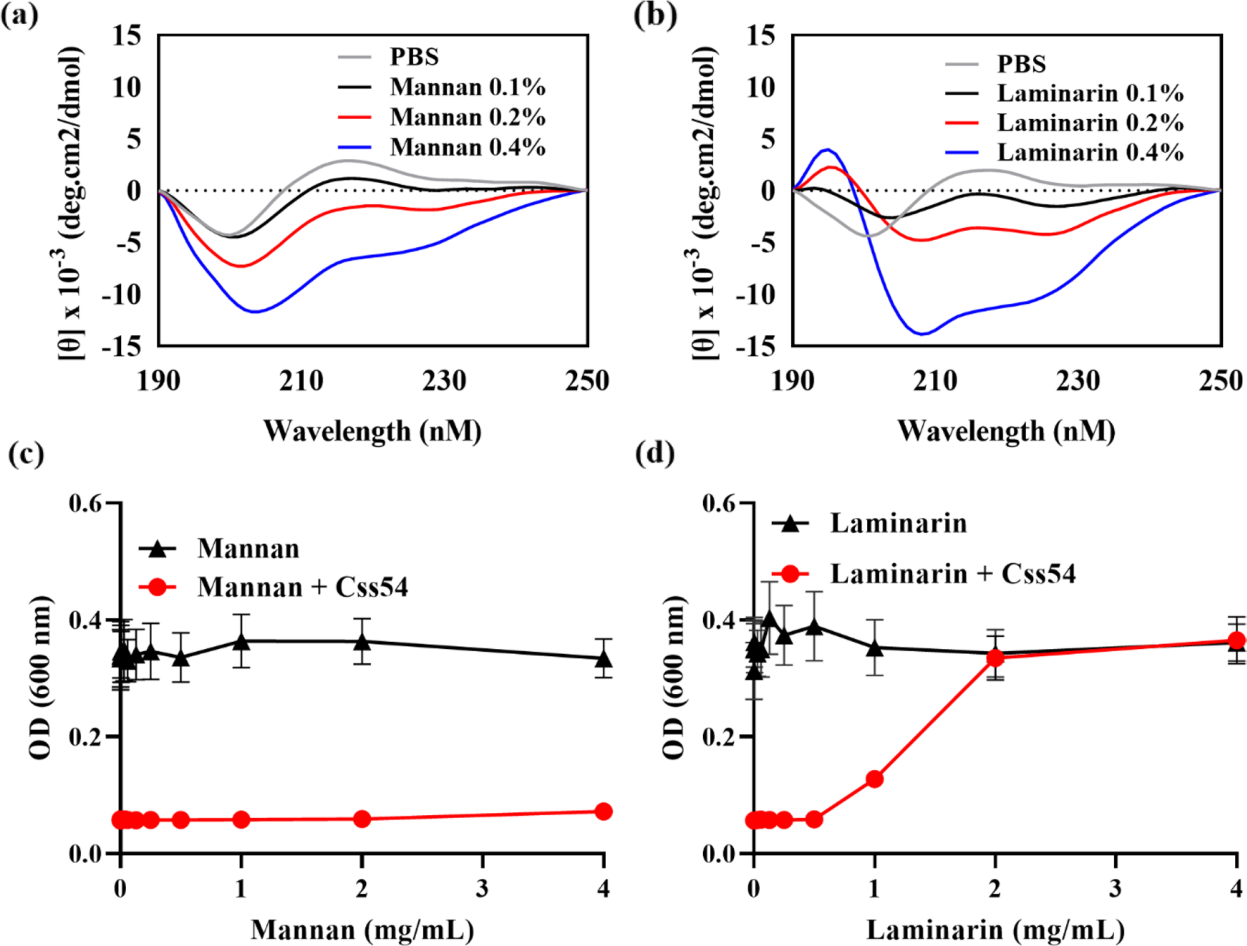
Analysis of the interaction between Css54 and cell wall components. CD spectra for Css54 at a concentration of 40 μM were obtained in PBS and co-incubated with **A** mannan or **B** laminarin. The antifungal activity of Css54 against *C. albicans* was investigated in the presence of various concentrations of **C** mannan and **D** laminarin from 0 to 4 mg/ml. Data are shown as mean ± SEM (n = 6)

CD spectroscopy was used to assess the structure of Css54 in the presence of polysaccharides from fungal membranes such as mannan and laminarin. In aqueous solution, Css54 exhibited a random coil structure. Even in the presence of mannan, Cssd54 exhibited a random-coil structure (Fig. 3A). However, in presence of laminarin, Css54 adopted the α-helix structure (Fig. 3B). To confirm these results, we examined the antifungal activity of Css54 in the presence of mannan and laminarin. Css54 retained its antifungal activity even in the presence of mannan (Fig. 3C); however, the antifungal activity of Css54 decreased in the presence of laminarin at 1 mg/ml (Fig. 3D). These results demonstrated that Css54 binds to laminarin, a fungal membrane component, and affects its antifungal activity.

### Membrane Depolarization of *C. albicans*

**Fig. 4:**
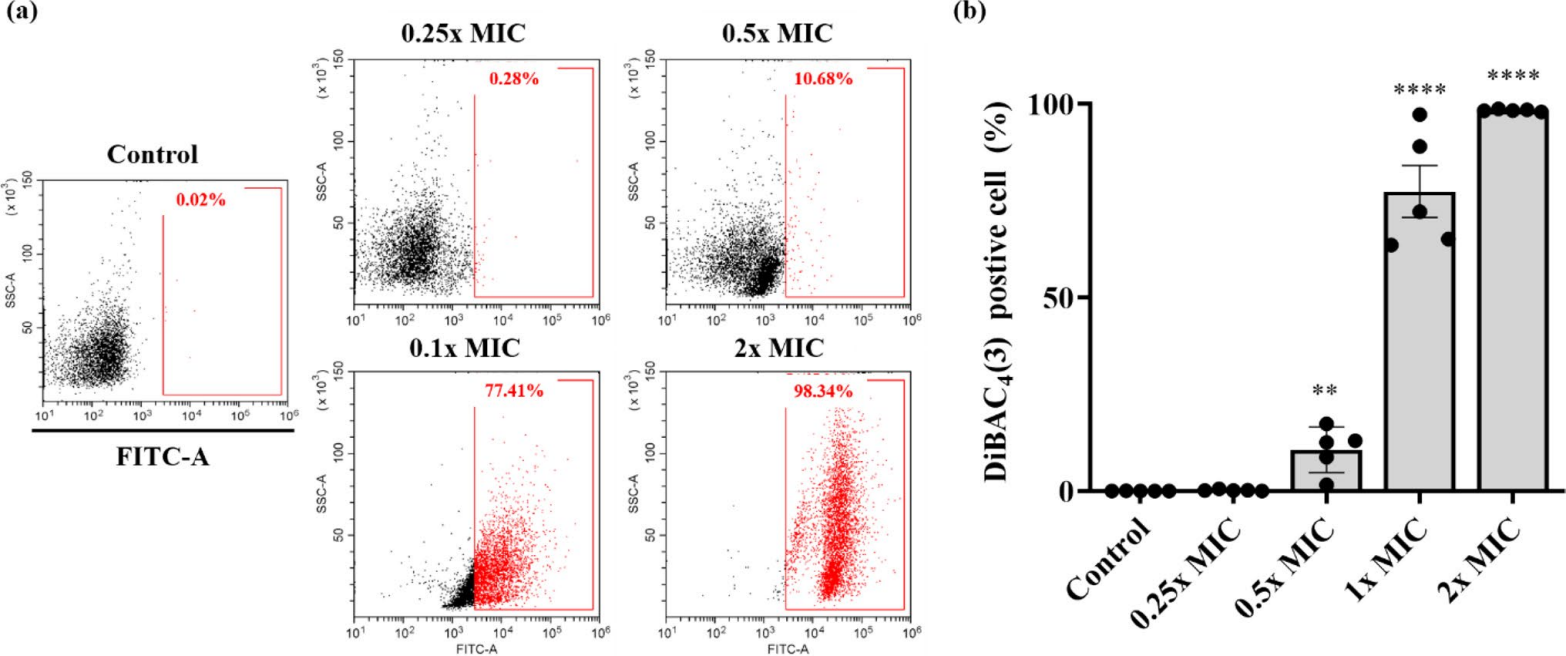
Observation of the membrane potential of *C. albicans* following Css54 treatment. DiBAC_4_(3) was used to detect change in membrane potential. **A** Alternations in membrane depolarization of *C. albicans* after exposure to Css54 at 0.25 × , 0.5 × , 1 × , and 2 × MICs using flow cytometry. **B** The statistical analyses are presented as averages. Data are shown as mean ± SEM (n = 5). *P < 0.05, **P < 0.01, ***P < 0.001, **** *P* < 0.0001 versus control (unpaired *t-test*)

The effect of Css54 on *C. albicans* membrane potential was analyzed using DiBAC_4_(3), a fluorescent dye that penetrates cells via depolarized membranes and binds to intracellular components or membranes (Buakaew et al., 2022). *Candida albicans* was exposed to Css54 at concentrations of 0.25 × , 0.5 × , 1 × , and 2 × MICs. Only 0.02% of stained *C. albicans* stain positive for DiBAC_4_(3). However, in *C. albicans* treated with Css54 at 0.25 × , 0.5 × , 1 × , and 2 × MICs, the fluorescence intensities were 0.28%, 10.68%, 77.41%, and 98.34%. Compared to the control, Css54 increased the fluorescence intensity of DiBAC_4_(3) in a dose-dependent manner (Fig. 4A, B).

### Impact of Css54 on the Membrane Integrity of *C. albicans*

**Fig. 5:**
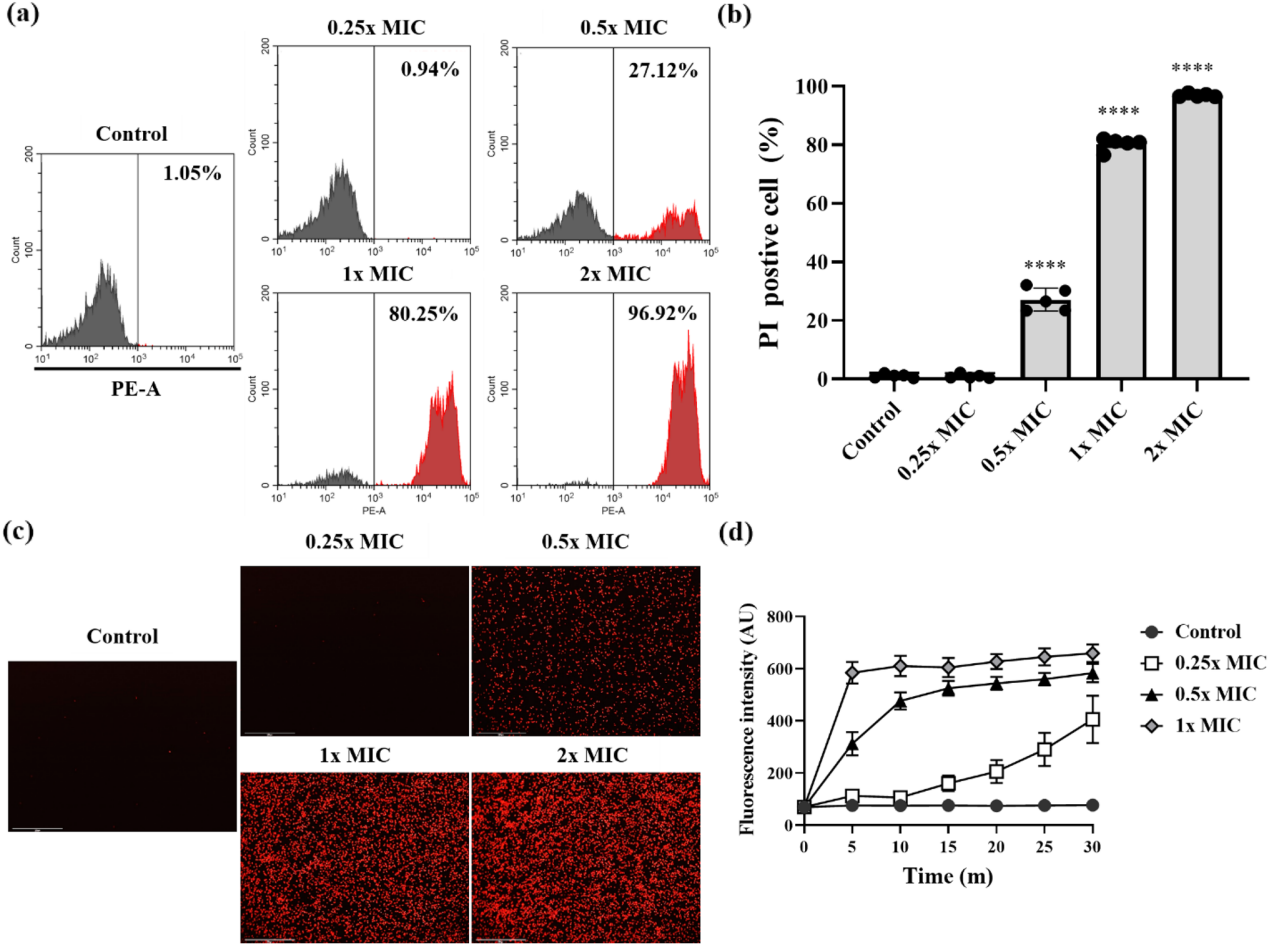
Investigation of *C. albicans* membrane integrity after exposure to Css54. **A** After treatment with Css54 at 0.25 × , 0.5 × , 1 × , and 2 × MICs and staining with PI, the membrane integrity of *C. albicans* was confirmed using flow cytometry. **B** The statistical analyses are presented as averages (*n* = 5). **C** Fluorescence microscopy of *C. albicans* stained with PI after incubation with Css54 at 0.25 × , 0.5 × , 1 × , and 2 × MICs for 10 min. **D** Evaluation of *C. albicans* membrane integrity after exposure to Css54 using SYTOX green were analyzed *C. albicans* loaded with SYTOX green were treatment with Css54 at 0.25 × , 0.5 × , 1 × , and 2 × MICs. Data are shown as mean ± SEM (n = 5) *P < 0.05, **P < 0.01, ***P < 0.001, **** *P* < 0.0001 versus control (unpaired *t-test)*

We next evaluated the effect of Css54 on the fungal membrane integrity using PI and SYTOX Green, both of which can penetrate compromised membranes and bind to nucleic acids, resulting in increased fluorescence intensity (Vaessen et al., 2019). Initially, we employed PI and flow cytometry to evaluate whether Css54 disrupts the membrane of *C. albicans*. In the control groups, only 1.7% of *C. albicans* was stained with PI, suggesting preserved membrane integrity. However, after treatment with Css54 at 0.25 × , 0.5 × , 1 × , and 2 × MICs, PI-positive *C. albicans* increased to 0.9%, 027.12%, 80.25%, and 96.92%, respectively (Fig. 5A, B). Moreover, we used fluorescence microscopy to visualize PI in *C. albicans* after exposure to Css54. We also found that Css54 treatment of *C. albicans* increased the fluorescence intensity of PI in a time-dependent manner (Fig. S2A and S2B). Finally, we used fluorescence microscopy to visualize the PI and confirmed that Css54 increased the fluorescence intensity in *C. albicans* in a time- and concentration-dependent manner (Fig. 5C). These results were demonstrated using a SYTOX green uptake assay. Css54 increased the fluorescence intensity of SYTOX Green in a time- and concentration-dependent manner (Fig. 5D). These results suggested that Css54 exhibits antifungal activity by affecting the membrane of *C. albicans*.

### Measurement of ROS Production

**Fig. 6:**
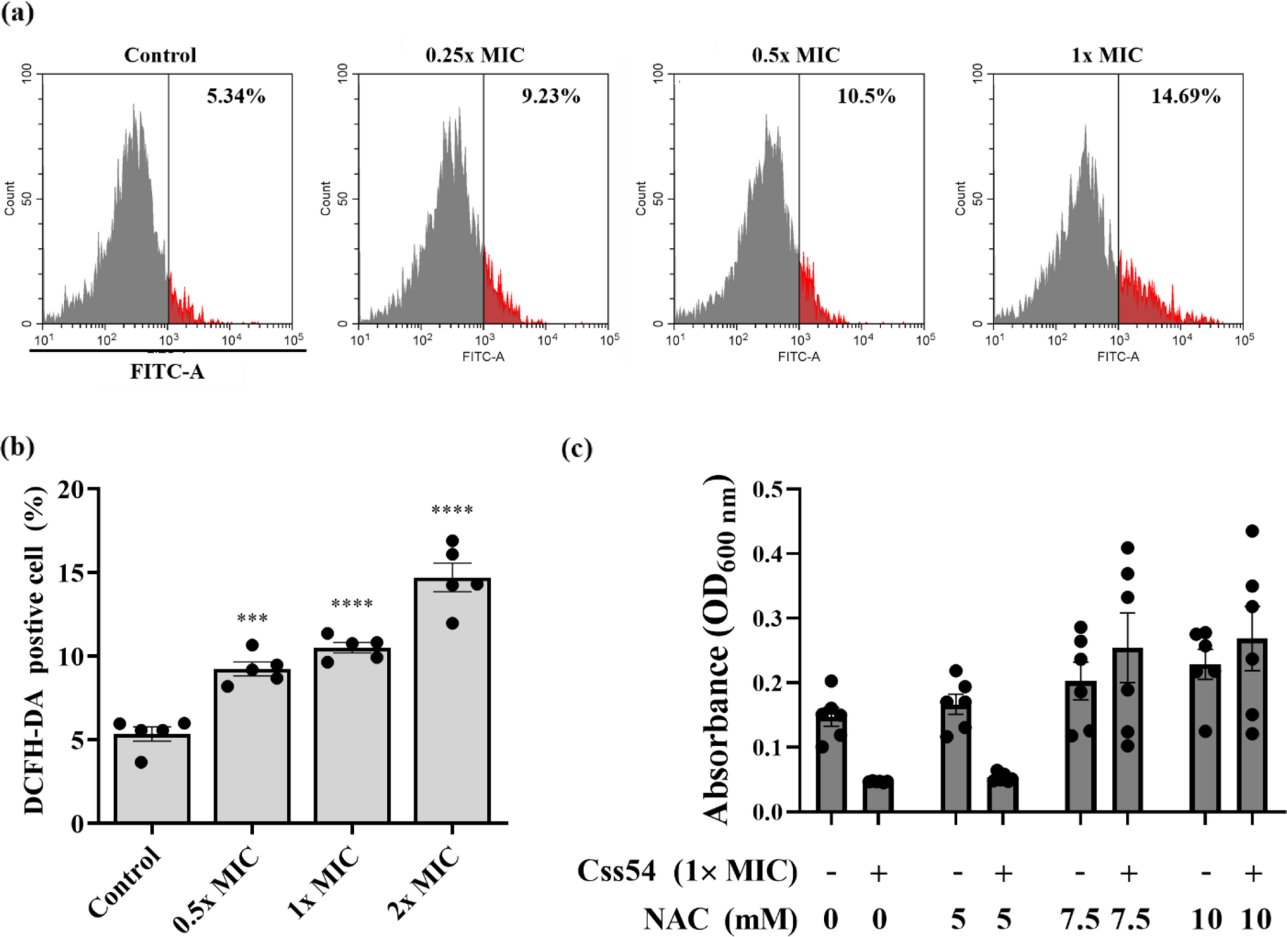
Effects of Css54 on ROS generation in *C. albicans*. **A** Using flow cytometry to DCFH-DA detection, ROS production was measured in the presence of Css54 at 0.25 × , 0.5 × , 1 × , and 2 × MICs added to *C. albicans*, The antifungal activity of Css54 against *C. albicans* was investigated in presence of various concentrations of NAC at 5, 7.5, 10 mM. **B** The statistical analyses are presented as averages. Data are shown as mean ± SEM (n = 5) *P < 0.05, **P < 0.01, ***P < 0.001, **** *P* < 0.0001versus control (unpaired *t-test*). **C** Antifungal activity of Css54 against *C. albicans* in the presence of varying concentrations of NAC. Data are shown as mean ± SEM (n = 6)

We next used DCFH-DA, a fluorescent probe, to evaluate the induction of intracellular ROS in *C. albicans* after treatment with Css54 (Seyedjavadi et al., 2020). The original DCFH-DA is non-fluorescent; in the presence of ROS, DCFH-DA is cleaved to produce DCFH, which is then oxidized to DCF, a fluorescent dye (Liu et al., 2022). In the control group, 5.34% of *C. albicans* showed ROS-positive staining. DCF fluorescence increased significantly after 2 h of incubation with Css54. In response to Css54 at 2 × MIC, 14.69% of the *C. albicans* exhibited ROS-positive staining. Even at concentrations below the 2 × MIC, ROS-positive staining increased in a concentration-dependent manner compared with the control (Fig. 6A, B). Furthermore, to determine whether Css54 exhibits antifungal activity through ROS production, we suppressed ROS generation using NAC as an ROS inhibitor (Fig. 6C). Css54 exhibited reduced antifungal activity in an NAC concentration-dependent manner. These results demonstrate that Css54 induces ROS generation, which causes oxidative damage to the *C. albicans* membrane.

## Discussion

In many organisms, innate defense mechanisms involve the production of AMPs, which have recently emerged as a significant source of antimicrobial drugs. To address antifungal resistance, new antifungal agents have been developed using AMPs isolated from a wide variety of organisms to overcome this issue. The mode of action of AMPs involves membrane disruption and targeting of intracellular components, inhibiting essential microbial metabolic functions, and thus showing minimal correlation with antimicrobial resistance (Rincón-Cortés et al., 2022).

Nature has provided compounds for the treatment of various diseases. Natural antimicrobials, studied as potential alternatives to commercial antimicrobials, are crucial because of the increasing resistance issues (Yacoub et al., 2020). Venomous animals have specialized apparati for toxin delivery, which can be used defensively or for immobilization. Researchers have long been interested in animal venoms as potential sources of therapeutic compounds for the treatment of specific diseases (Lamiyan et al., 2020). Animal venoms contain antimicrobial molecules, which are potential leads to development of novel therapeutic agents. AMPs, prevalent in scorpion venom, have been identified as potential alternatives to traditional venom antibiotics (Silva et al., 2020).

In 2013, Gracia et al. isolated Css54 (of 25 amino acids) from *C. s. suffusus* of scorpions. Css54 typically has a cationic structure consisting of lysine and hydrophobic amino acids, such as isoleucine and leucine, which confer a distinct α-helical structure. Css54 shows antimicrobial activity against *E. coli* and *S. aureus* (Garcia et al., 2013). In this study, we investigated the antimicrobial activity of Css54 against bacteria associated with zoonotic diseases. We confirmed that the Css54 peptide exerted bactericidal activity by perturbing the bacterial membrane. Melittin, a 26-amino-acid peptide extracted from bee venom, was used as a positive control peptide due to its established role as a representative lytic peptide with potent antimicrobial efficacy (Sun et al., 2022). In vivo studies have established melittin as a promising therapeutic agent with significant anti-cancer (Dabbagh Moghaddam et al., 2021) and anti-inflammatory activities (Park et al., 2012). Relative to melittin, Css54 exhibited reduced hemolytic activity. Furthermore, Css54 exhibits lower cytotoxicity than melittin in PK(15) cells (a cell line isolated from porcine kidneys) (Park et al., 2020).

However, as the antifungal activity of Css54 and its mechanism of action remain unclear, we aimed to elucidate these in the current study. Fungal infections are a significant public health challenge that contribute to a global increase in antimicrobial resistance (Fisher et al., 2022). Globally, *Candida* species are responsible for the majority of opportunistic fungi and are associated with high rates of mortality and morbidity. *Candida albicans* is the predominant pathogen implicated in candidemia. Invasive fungal diseases in humans are primarily caused by *C. albicans* and are associated with a high mortality rate. *C. albicans* is the most common causative agent of candidiasis (Seyoum et al., 2020). In recent years, *C. albicans* has become increasingly resistant to traditional antifungal agents, posing a great challenge for clinical treatment (Costa-de-Oliveira & Rodrigues, 2020). Antifungal agents, classified as azoles, echinocandins, or polyenes, are limited compared to antibacterial drugs (Tan et al., 2018). Therefore, novel antifungal agents are required to effectively combat *Candida* infections.

This study demonstrated that Css54 effectively inhibit growth of *C. albicans* at a concentration of 2 μM to 4 μM. CSS54 completely killed *C. albicans* within 6 h at 2 × MIC. A key virulence factor of *Candida albicans* is its ability to establish biofilms and form cell aggregates on surfaces. Biofilm formation by *C. albicans* enhances its resistance to antimicrobial agents and facilitates evasion of the host immune response, complicating therapeutic interventions (Gulati & Nobile, 2016). Therefore, we used crystal violet and SYTO9 staining to confirm that Css54 inhibits biofilms formed by *C. albicans*. Css54 inhibited 90% of *C. albicans* biofilm formation at 4 μM. These results are of particular interest owing to the increasing prevalence of antifungal-resistant strains and the limited selection of antifungal agents available.

Initially, AMPs bind to fungal cell membranes, either by disrupting them or by interacting with intracellular targets. AMPs that bind to the cell wall components of *C. albicans* are important for its antifungal activities (Jang et al., 2010). *Candida albicans* cell wall of *C. albicans* plays a critical role in interacting with its environment and host. To investigate the antifungal properties of CSS54, we determined the components of the cell wall of *C. albicans* that bind to it. The *C. albicans* cell wall is predominantly composed of carbohydrates, notably mannan, ß-1,6-glucan, and chitin. In the cell wall of *C. albicans*, mannan is covalently linked to proteins, accounting for 40% of the total polysaccharides (Tsai et al., 2011). Laminarin is primarily composed in ß-1,3-glucan. In the present study, we established that Css54 interacts with *C. albicans* cell wall carbohydrates, specifically laminarin, which inhibits its antifungal activity of Css54. Css54 had a random coil structure in the presence of mannan; however, in the presence of laminarin, the secondary structure changed to an alpha helix (Fig. 3). These results suggest that laminarin plays an important role in the antifungal activity of Css54.

We investigated the antifungal mechanism of Css54 against *C. albicans*. We used fluorescent dyes to determine whether Css54 affects the *C. albicans* membrane. First, the mode of action of AMPs can be mediated by the depolarization of the normally polarized membrane (Lee et al., 2015). Using DiBAC_4_(3), a membrane potential indicator, we confirmed that Css54 increased the membrane depolarization of *C. albicans* (Fig. 4). Based on the observed increase in membrane depolarization, we hypothesized that Css54 exhibited antifungal activity through a membrane-targeting mechanism. PI and SYTOX Green were used to assess cell membrane integrity. Using flow cytometry and fluorescence spectrophotometry, we observed elevated uptake of fluorescence intensity of both dyes in Css54 exposed *C. albicans* compared to that in untreated controls. Following treatment with Css54, fluorescence microscopy revealed an increase in PI uptake in a concentration- and time-dependent manner (Fig. 5). Damage to the membrane integrity in yeast has been reported to be associated with excessive ROS production. Damage to the membrane integrity in yeast has been reported to be associated with excessive ROS production. ROS induce the production of polar lipid hydroperoxides, which disrupt the bilayer membrane structure and modify its properties. In this study, DCFH-DA staining was used to observe whether ROS levels were increased in the presence of Css54. We used the ROS inhibitor NAC to determine whether Css54-induced ROS production in *C. albicans* affected its antifungal activity. We found that the presence of NAC reduced the antifungal activity of Css54 (Fig. 6). These findings suggest that increased ROS generation following Css54 exposure influences the antifungal activity of *C. albicans*.

In conclusion, Css54 was identified as a potential alternative antifungal agent. Css54 exhibits antifungal activity against *C. albicans*. In addition, Css54 effectively inhibits biofilm formation by *C. albicans*. We demonstrated that Css54 binds to laminarin based on the CD spectra. After binding laminarin, Css54 disrupts the *C. albicans* membrane. Furthermore, we found that Css54 induced ROS in *C. albicans* affected its antifungal activity. As a result of our research, Css54 may be useful as an alternative therapy against infection caused by *C. albicans*.

### Supplementary Information

Below is the link to the electronic supplementary material.

Supplementary file1 (PDF 314 KB)

## Data Availability

The supporting data for this study′s findings are available from the corresponding authors upon reasonable request.
