# Supplementary material for: Exploring the Therapeutic Potential of Scorpion-Derived Css54 Peptide Against Candida albicans
Source: J Microbiol. 2024 Apr 8;62(2):101–12. doi: 10.1007/s12275-024-00113-4 (PMC11021323; doi:10.1007/s12275-024-00113-4)
Supplement: Supplementary file 1 — Supplementary file1 (PDF 314 KB) [file 12275_2024_113_MOESM1_ESM.pdf]

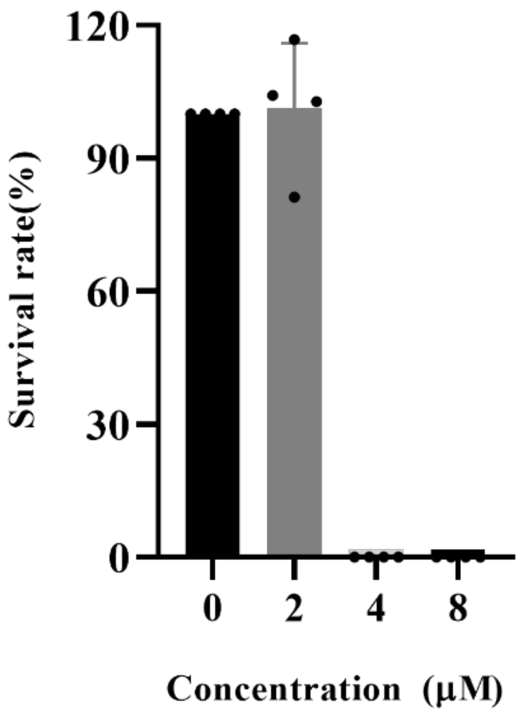

Fig. S1. Antifungal activity of Css54 against *C. albicans* KCTC 7270 in RPMI. Colony counts of *C. albicans* from different groups. Survival rate (%) Control group/statistical analyses are presented as averages (n = 4). *C. albicans* survival rate (%) = (number of colonies treated with Css54/number of colonies untreated control) × 100.

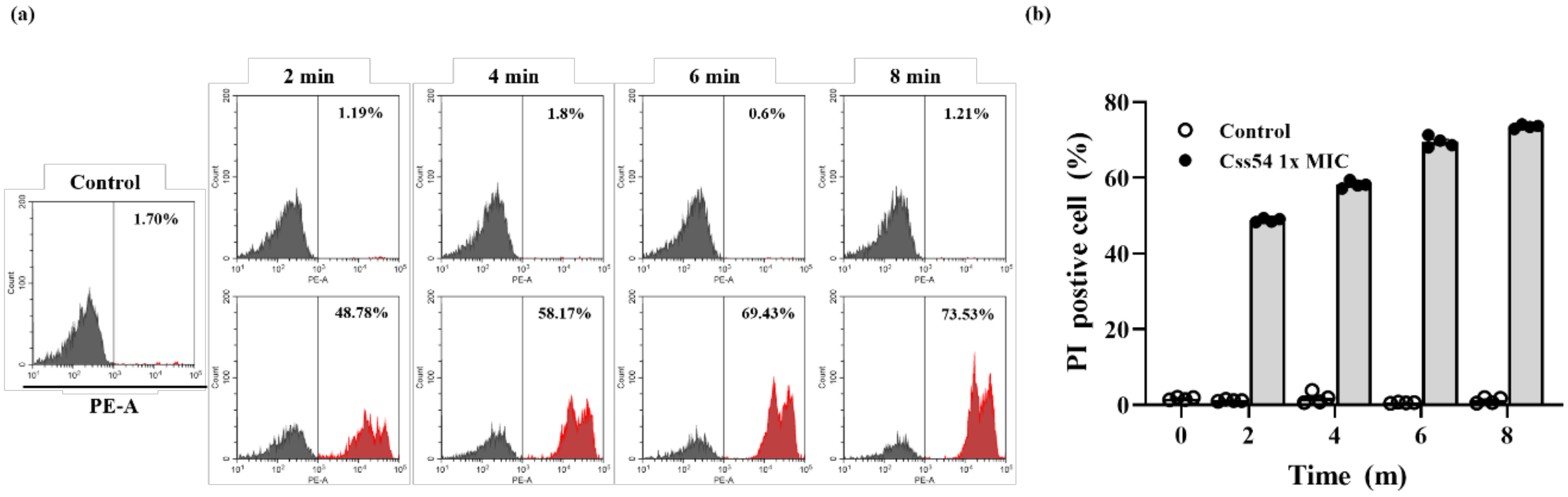

Fig. S2. Analysis of *C. albicans* membrane integrity after exposure to Css54. (A) PI staining for 8 min at 2 min intervals after treatment with Css54 to *C. albicans* were confirmed using flow cytometry. (B) The statistical analyses are presented as averages ($n = 4$).
